# Pseudoalteromone B: A Novel 15C Compound from a Marine Bacterium *Pseudoalteromonas* sp. CGH2XX 

**DOI:** 10.3390/md10071566

**Published:** 2012-07-20

**Authors:** Yu-Hsin Chen, Jimmy Kuo, Jui-Hsin Su, Tsong-Long Hwang, Yung-Husan Chen, Chia-Hung Lee, Ching-Feng Weng, Ping-Jyun Sung

**Affiliations:** 1 Department of Life Science and Institute of Biotechnology, National Dong Hwa University, Hualien 974, Taiwan; Email: kb5634@yahoo.com.tw (Y.-H.C.); chlee016@mail.ndhu.edu.tw (C.-H.L.); 2 Graduate Institute of Marine Biotechnology, National Dong Hwa University, Pingtung 944, Taiwan; Email: jimmy@nmmba.gov.tw (J.K.); x2219@nmmba.gov.tw (J.-H.S.); 3 National Museum of Marine Biology and Aquarium, Pingtung 944, Taiwan; Email: tony_chen72001@yahoo.com.tw; 4 Graduate Institute of Natural Products, Chang Gung University, Taoyuan 333, Taiwan; Email: htl@mail.cgu.edu.tw; 5 Department of Marine Biotechnology and Resources and Division of Marine Biotechnology, Asia-Pacific Ocean Research Center, National Sun Yat-sen University, Kaohsiung 804, Taiwan

**Keywords:** pseudoalteromone, *Pseudoalteromonas*, anti-inflammatory, *Lobophytum crassum*, elastase

## Abstract

A novel 15C compound, pseudoalteromone B (**1**), possessing a novel carbon skeleton, was obtained from a marine bacterium *Pseudoalteromonas* sp. CGH2XX. This bacterium was originally isolated from a cultured-type octocoral *Lobophytum crassum*, that was growing in cultivating tanks equipped with a flow-through sea water system. The structure of **1** was established by spectroscopic methods. Pseudoalteromone B (**1**) displayed a modestly inhibitory effect on the release of elastase by human neutrophils.

## 1. Introduction

**Figure 1:**
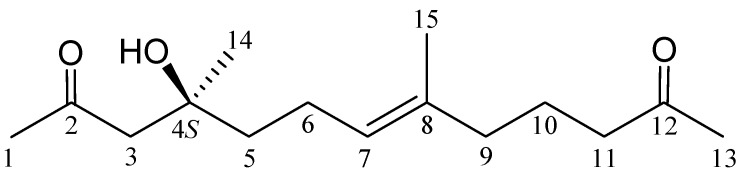
The structure of pseudoalteromone B (**1**).

Marine bacteria belonging to the genus *Pseudoalteromonas* sp. (family Pseudoalteromonadaceae) have proven to be not only an important source of various antibiotics, but have also played an interesting role in marine ecology [1,2,3,4]. In the continuing research aimed at the discovery of new natural substances from marine microorganisms, an organic extract of the bacterium identified as *Pseudoalteromonas* sp. CGH2XX, which was originally isolated from a cultured-type octocoral *Lobophytum crassum* (family Alcyonacea), exhibited significant cytotoxicity toward the HL-60 (human acute promyelocytic leukemia) and CCRF-CEM (human T cell acute lymphoblastic leukemia) tumor cells (IC_50_ = 0.9, 1.2 µg/mL) and displayed a significant inhibitory effect (inhibition rate 45.1%) on the release of elastase by human neutrophils at a concentration of 10 µg/mL. We isolated a novel 15C compound, pseudoalteromone B (**1**) (Figure 1), from this microorganism. The structure of **1** was established by spectroscopic methods and this compound displayed a modestly inhibitory effect on the release of elastase by human neutrophils.

## 2. Results and Discussion

Pseudoalteromone B (**1**) was isolated as an oil and had the molecular formula C_15_H_26_O_3_, as determined by HRESIMS (C_15_H_26_O_3_ + Na, *m/z* found 277.1779, calculated 277.1780) indicating three degrees of unsaturation. The IR absorption bands at 3502 and 1706 cm^−1^ were characteristic for the hydroxy and ketone groups. 

**Table 1:** ^1^H (400 MHz, CDCl_3_) and ^13^C (100 MHz, CDCl_3_) NMR data for **1**.

Position	*δ*_H_ (*J* in Hz)	*δ*_C,_ Mult.
1	2.18 s	31.9, CH_3_
2		211.0, qC
3a/b	2.58 d (17.2); 2.65 d (17.2)	52.3, CH_2_
4		71.5, qC
5	1.51 m	41.9, CH_2_
6	2.04 m	22.5, CH_2_
7	5.09 tq (7.2, 1.2)	124.8, CH
8		134.6, qC
9	1.96 t (7.2)	38.8, CH_2_
10	1.66 quintet (7.2)	21.8, CH_2_
11	2.37 t (7.2)	43.0, CH_2_
12		209.1, qC
13	2.12 s	29.9, CH_3_
14	1.22 s	26.7, CH_3_
15	1.58 br s	15.7, CH_3_

The ^1^H and ^13^C NMR data of **1** (Table 1) showed the presence of 15 carbon signals, which were identified by the assistance of a DEPT spectrum as four methyls, six sp^3^ methylenes, an sp^2^ methine, an sp^3^ quaternary carbon, and three sp^2^ quaternary carbons including two ketone carbonyls. The ^1^H NMR spectrum of **1** showed a signal of olefinic proton (*δ*_H_ 5.09, 1H, tq, *J* = 7.2, 1.2 Hz, H-7), two acetyl methyls (*δ*_H_ 2.18, 3H, s, H_3_-1; 2.12, 3H, s, H_3_-13), a vinyl methyl (*δ*_H_ 1.58, 3H, br s, H_3_-15), a tertiary methyl attaching at an oxygenated quaternary carbon (*δ*_H_ 1.22, 3H, s, H_3_-14) and six pairs of methylene protons (*δ*_H_ 2.65, 1H, d, *J* = 17.2 Hz; 2.58, 1H, d, *J* = 17.2 Hz, H_2_-3; 2.37, 2H, t, *J* = 7.2 Hz, H_2_-11; 2.04, 2H, m, H_2_-6; 1.96, 2H, t, *J* = 7.2 Hz, H_2_-9; 1.66, 2H, quintet, *J* = 7.2 Hz, H_2_-10; 1.51, 2H, m, H_2_-5).

**Figure 2:**
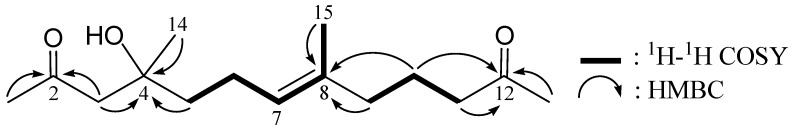
The ^1^H–^1^H COSY and selective HMBC correlations (protons→quaternary carbons) of **1**.

The constitution of the carbon skeleton of **1** was elucidated initially by the ^1^H–^1^H COSY and HMBC correlations of **1** (Figure 2), it was possible to establish the separate spin systems that map out the proton sequences from H_2_-5/H_2_-6/H-7 and H_2_-9/H_2_-10/H_2_-11. These data, together with the HMBC correlations between H_3_-1/C-2, C-3; H_2_-3/C-2, C-4, C-5; H_2_-5/C-4, C-6; H-7/C-9; H_2_-9/C-7, C-8, C-10, C-11; H_2_-10/C-8, C-9, C-11, C-12; H_2_-11/C-9, C-10, C-12; and H_3_-13/C-11, C-12, permitted elucidation of the main straight carbon skeleton. The vinyl methyl at C-8 was confirmed by the HMBC correlations between H-7, H_2_-9/C-15; and H_3_-15/C-7, C-8, C-9; and further supported by an allylic coupling between H-7 and H_3_-15 (*J* = 1.2 Hz). Based on these data, together with the HMBC correlations between H_3_-14/C-3, C-4, C-5 and H_2_-3, H_2_-5/C-14, the planar structure of **1** was established.

**Figure 3:**
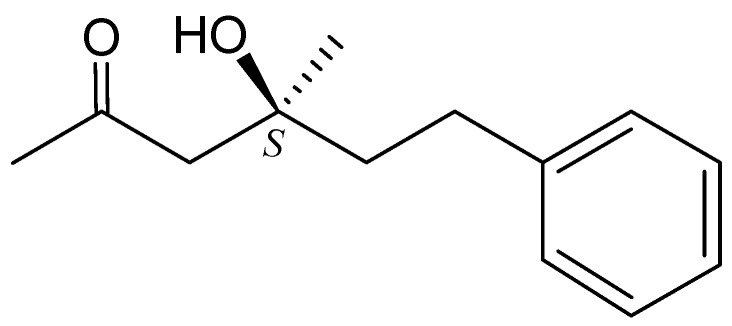
The structure of (*S*)-4-hydroxy-4-methyl-6-phenylhexan-2-one (**2**).

In the NOESY experiment of **1**, a correlation between H-7 with H_2_-9, as well as the lack of correlation between H-7 and H_3_-15, reflected the *E*-configuration of C-7/8 double bond. Furthermore, by comparison of the rotation value of **1** ([α]^23^_D_ −20 (*c* 0.03, CHCl_3_)) with that of a known synthetic compound, (*S*)-4-hydroxy-4-methyl-6-phenylhexan-2-one (**2**) ([α]^25^_D_ −14.5 (*c* 1.1, CHCl_3_)) (Figure 3) [5], the absolute configuration for the C-4 chiral center of **1** was determined as *S* form as that of **2**. Based on the above findings, the structure of **1** was determined unambiguously.

The *in vitro* cytotoxicity of pseudoalteromone B (**1**) toward HCT116 (human colorectal carcinoma), K-562 (human chronic myelogenous leukemia), HL-60 (human acute promyelocytic leukemia), CCRF-CEM (human T cell acute lymphoblastic leukemia), T-47D (human breast ductal carcinoma), and MDA-MB-231 (human breast adenocarcinoma) cells was tested. Unfortunately, the new compound **1** described herein is not active toward the above cells (all IC_50_ values > 20 µg/mL). The *in vitro* anti-inflammatory effect of **1** was tested. Pseudoalteromone B (**1**) displayed a modestly inhibitory effect (inhibition rate 20.7%) on the release of elastase by human neutrophils at a concentration of 10 µg/mL.

## 3. Experimental Section

### 3.1. General Experimental Procedures

Optical rotations were measured on a Jasco P-1020 polarimeter. IR spectra were recorded on a Jasco FT/IR-4100 infrared spectrophotometer. The NMR spectra were recorded on a Varian Mercury Plus 400 FT-NMR at 400 MHz for ^1^H and 100 MHz for ^13^C, in CDCl_3_, respectively. Proton chemical shifts were referenced to the residual CHCl_3_ signal (*δ*_H_ 7.26 ppm). ^13^C NMR spectra were referenced to the center peak of CDCl_3_ at *δ*_C_ 77.1 ppm. ESIMS and HRESIMS data were recorded on a Bruker APEX II mass spectrometer. Silica gel (Merck, 230–400 mesh) and Sephadex LH-20 (Amersham Biosciences) were used for column chromatography. TLC was carried out on precoated Kieselgel 60 F_254_ (0.25 mm, Merck); spots were visualized by spraying with 10% H_2_SO_4_ solution followed by heating.

### 3.2. Marine Bacteria Isolation, Culture Conditions and Extract Preparation

A marine bacterium number CGH2XX was isolated from soft coral *Lobophytum crassum* that was growing in cultivating tanks equipped with a flow-through sea water system [4]. The bacterium strain CGH2XX was 98.3% identical with *Pseudoalteromonas* sp. H02P24-23 (Genebank accession no. HQ161380) on the basis of 16S rDNA gene sequence. The marine bacterium was cultured in 2.5 L flasks containing 1 L M1 broth (not containing agar) with 80% seawater. Flasks were incubated at 25 °C on a rotatory shaker at 120 rpm. After five days of incubation, extraction of the culture broth (10.0 L) with ethyl acetate (EtOAc, 2 × 10.0 L) yielded 1.71 g of crude extract. The extracts obtained were stored at −20 °C.

### 3.3. Separation

Crude extract was separated on Sephadex LH-20 and eluted using a mixture of dichloromethane and methanol (1:1) to yield 17 fractions. Fraction 6 was selected for further study and purified by silica gel, using a mixture of *n*-hexane and EtOAc (2:1) as a mobile phase to afford compound **1** (4.2 mg).

Pseudoalteromone B (**1**): colorless oil; [α]^23^_D_ −20 (*c* 0.03, CHCl_3_); IR (neat) ν_max_ 3502, 1706 cm^−1^; ^1^H (CDCl_3_, 400 MHz) and^ 13^C (CDCl_3_, 100 MHz) NMR data, see Table 1; ESIMS: *m/z* 277 (M + Na)^+^; HRESIMS: *m/z* 277.1779 (calcd for C_15_H_26_O_3_ + Na, 277.1780).

### 3.4. Cytotoxicity Testing

The cytotoxicity of compound **1 **was assayed with a modification of the MTT [3-(4,5-dimethylthiazol-2-yl)-2,5-diphenyltetrazolium bromide] colorimetric method. Cytotoxicity assays were carried out according to previously described procedures [6,7,8].

### 3.5. Elastase Release by Human Neutrophils

Human neutrophils were obtained by means of dextran sedimentation and Ficoll centrifugation. Elastase release experiments were performed using MeO-Suc-Ala-Ala-Pro-Valp-nitroanilide as the elastase substrate [9,10,11].

## 4. Conclusions

In a previous study [4], an ubiquinone derivative, pseudoalteromone A, was isolated from *Pseudoalteromonas* sp. CGH2XX, and this compound was found to be cytotoxic toward MOLT-4 (human acute lymphoblastic leukemia) and T-47D (human breast ductal carcinoma) cells (IC_50_ = 3.8, 4.0 µg/mL) and displayed moderately inhibitory effects on the generation of superoxide anion and the release of elastase (inhibition rates 38.0, 20.2%) by human neutrophils at a concentration of 10 µg/mL [12]. However, as described in the beginning of this communication, the organic extract of *Pseudoalteromonas* sp. CGH2XX showed significant cytotoxicity and anti-inflammatory activity. At this stage, the results showed that pseudoalteromone B (**1**) displayed a modestly anti-inflammatory activity and this compound was not cytotoxic toward HCT116, K-562, HL-60, CCRF-CEM, T-47D and MDA-MB-231 cells. We suggested that the other active components exist in the other fractions. The possible activity for pseudoalteromone B (**1**) will be studied if we can get enough material from *Pseudoalteromonas* sp. CGH2XX. Furthermore, to the best of our knowledge, compounds pseudoalteromones A and B, were the first two compounds from the marine bacterium belonging to the genus *Pseudoalteromonas* associated with octocorals.
